# Efficacy of psychotherapies for mental disorders

**DOI:** 10.1192/j.eurpsy.2026.10406

**Published:** 2026-07-31

**Authors:** P. Cuijpers

**Affiliations:** WHO Collaborating Centre for Research and Dissemination of Psychological Interventions, Vrije Universiteit Amsterdam, Amsterdam, Netherlands

## Abstract

**Abstract:**

Hundreds of randomized controlled trials have examined the effects of psychotherapies on mental disorders. In my presentation I will give an overview of the current knowledge about these effects, based on the Metapsy initiative, a large project with living systematic reviews of psychological treatments of mental disorders. Large meta-analyses of more than 1,000 randomized controlled trials show that psychotherapies are effective for many mental disorders, with substantial symptom reduction, meaningful response rates across age groups and diagnoses, and long-term effects. Cognitive behavioral therapy has been best examined, but there is no evidence in most disorders that it is more effective than other therapies. Although treatments work, population prevalence has not declined, and evidence-based care can only reduce the overall disease burden by roughly 40%. Innovations are therefore necessary, and many are currently developed. Four main domains of innovation will be highlighted: digital interventions, personalized treatments, development of new or improved therapies, and simplification and wider dissemination of existing therapies. However, there are no “silver bullets” that will dramatically change outcomes, and progress can only come from multiple small, incremental advances.

**Disclosure of Interest:**

None Declared

